# Human red blood cells express the RNA sensor TLR7

**DOI:** 10.1038/s41598-024-66410-5

**Published:** 2024-07-09

**Authors:** L. K. Metthew Lam, Emily Oatman, Kaitlyn A. Eckart, Nathan J. Klingensmith, Emily Flowers, Layal Sayegh, Julia Yuen, Rebecca L. Clements, Nuala J. Meyer, Kellie A. Jurado, Andrew E. Vaughan, Stephanie C. Eisenbarth, Nilam S. Mangalmurti

**Affiliations:** 1 Division of Pulmonary, Allergy, and Critical Care, Perelman School of Medicine at the University of Pennsylvania, Philadelphia, PA 19104 USA; 2 Division of Traumatology, Surgical Critical Care, and Emergency Surgical Services, Department of Surgery, Perelman School of Medicine at the University of Pennsylvania, Philadelphia, PA 19104 USA; 3 Department of Microbiology, University of Pennsylvania, Philadelphia, PA 19104 USA; 4 Department of Biomedical Sciences, University of Pennsylvania School of Veterinary Medicine, Philadelphia, PA 19104 USA; 5 Department Medicine, Division of Allergy and Immunology, Northwestern University Feinberg School of Medicine, Chicago, IL 60611 USA

**Keywords:** Red blood cell, Toll-like receptor, TLR7, RNA, Toll-like receptors, Viral infection, Sepsis

## Abstract

Red blood cells (RBCs) express the nucleic acid-binding toll-like receptor 9 (TLR9) and bind CpG-containing DNA. However, whether human RBCs express other nucleic acid-binding TLRs is unknown. Here we show that human RBCs express the RNA sensor TLR7. TLR7 is present on the red cell membrane and is associated with the RBC membrane protein Band 3. In patients with SARS-CoV2-associated sepsis, TLR7-Band 3 interactions in the RBC membrane are increased when compared with healthy controls. In vitro, RBCs bind synthetic ssRNA and RNA from ssRNA viruses. Thus, RBCs may serve as a previously unrecognized sink for exogenous RNA, expanding the repertoire of non-gas exchanging functions performed by RBCs.

## Introduction

Once regarded as immunologically inert oxygen carriers, red blood cells (RBCs) have recently been evidenced to actively participate in the innate immune response. They possess the capacity to either amplify or suppress the host's immune response to various stimuli through the release of reactive oxygen species (ROS) or the binding of chemokines, pathogens, and nucleic acids. Despite the absence of transcriptional and translational machinery, RBCs exhibit immune functionality by interacting with diverse inflammatory mediators via their expression of cell surface receptors, including but not limited to the Duffy Antigen receptor for chemokines (DARC), glycophorin A (GPA), complement receptor 1 (CR1), and Toll-like Receptor 9 (TLR9)^1^.

Nucleic acid-sensing is an essential feature of the immune response, critical for triggering downstream inflammation. The nucleic acid-sensing TLRs (TLR3, 7, 8, and 9) are primarily localized intracellularly in the vesicular system^2^. However, surface localization of these TLRs in specific cells facilitates cell-type-specific immune responses^3^. Although not previously known to be on erythrocytes, we have recently discovered the presence of TLR9 on the surface of mammalian erythrocytes^4,5^. Notably, the interaction between CpG-DNA and erythrocyte TLR9 offers significant advantages to the host, as it accelerates erythrophagocytosis and facilitates DNA delivery to immune cells^5,6^. Thus, erythrocytes emerge as early messengers, triggering critical inflammatory responses essential for host defense. Under basal conditions, RBCs were found to sequester CpG, serving as a physiological rheostat to maintain homeostasis by scavenging low levels of cell-free CpG-DNA^4^. However, during inflammatory states characterized by an excess of CpG-DNA, RBC binding accelerates erythrocyte senescence, marked by morphological alterations, loss of the self-marker CD47, and enhanced erythrophagocytosis^5,6^. While these discoveries shed light on the role of RBCs in immune responses, the expression of other TLRs by RBCs remains unknown^4,5^.

Cell-free RNA (cfRNA), comprising microRNA, long non-coding RNA, or fragments of mRNA, is found in circulation, most often enclosed within vesicles^7,8^. Different RNA types are known to associate with RBCs, prompting an inquiry into the interaction between RNA and mature, enucleated RBCs^9^. Here we report that human RBCs express the RNA sensor TLR7 and have the ability to bind exogenous viral RNA.

## Results

### RBCs express TLR7

**Figure 1:**
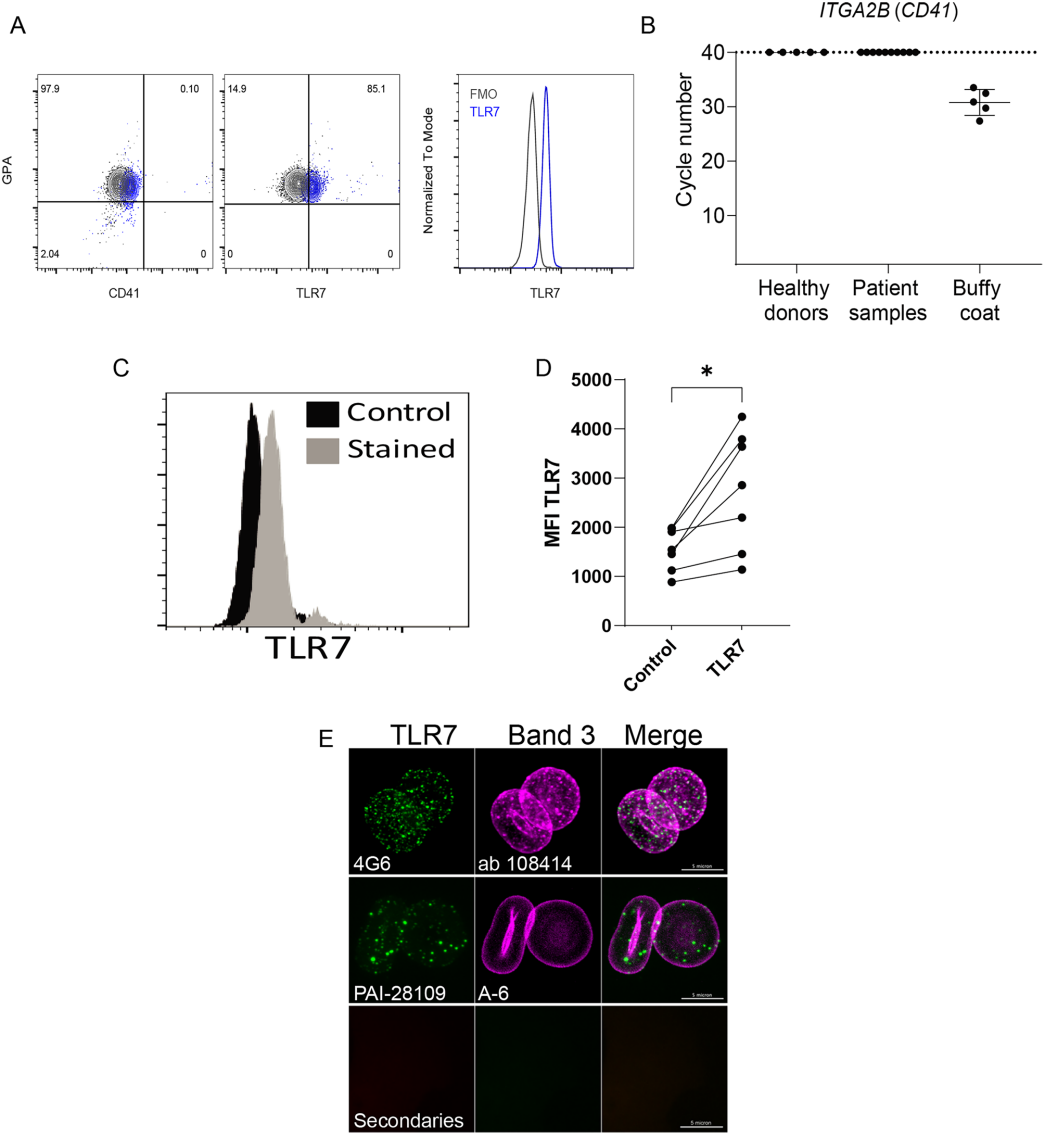
RBCs express TLR7 (**A**) GPA^+^/CD41^-^ cells express TLR7. Flow cytometry on purified RBCs from healthy donors was performed. RBCs were stained with GPA, CD41, and TLR7. GPA and CD41 staining is shown in the left panel, TLR7 staining on the GPA + cells is shown in the middle panel, histograms are provided in the right panel (**B**) qRT-PCR quantification of relative levels of *CD41* transcript in RBC preparations from healthy donors or patients with sepsis. Buffy coat was used as a positive control. RBC preparations are devoid of CD41. (**C**,**D**) Flow cytometry detection of TLR7 on RBCs from healthy donors. (**C**) Representative histograms of a healthy donor and (**D**) summarized data for geometric mean fluorescence intensity (GMFI), n = 7 donors, *P* = 0.02, paired t-test. (**E**) Confocal micrograph for immunofluorescent staining of TLR7 (green) and Band 3 (magenta) using two distinct pairs of antibodies; the antibody clone is indicated on the micrograph. Scale bar represents 5 μm.

Because we have previously identified the nucleic-acid sensing pattern recognition receptor (PRR) TLR9 on erythrocytes and *Tlr7* is expressed in erythroid precursors, we asked if TLR7 is present on RBCs^10^. TLR7 is expressed on human RBCs purified from whole blood of healthy donors (Fig. 1A). Using qRT-PCR to identify the platelet marker *Itga2b* (*CD41*), we verified that our RBC preparations were devoid of platelets, which also express TLR7 and TLR9 (Fig. 1B)^11,12^. We detected TLR7 expression on permeabilized RBCs (Fig. 1C,D) and confirmed our observations with confocal microscopy using two distinct antibody clones against TLR7 (clone 4G6 an PAI-2809), Fig. 1E. Surface TLR7 expression was evaluated using flow cytometry with an antibody clone against TLR7 (A94B10), we detected low levels of surface TLR7 expression on the RBC that appears to be heterogeneous across healthy donors (Supplemental Fig. 1).

**Figure 2:**
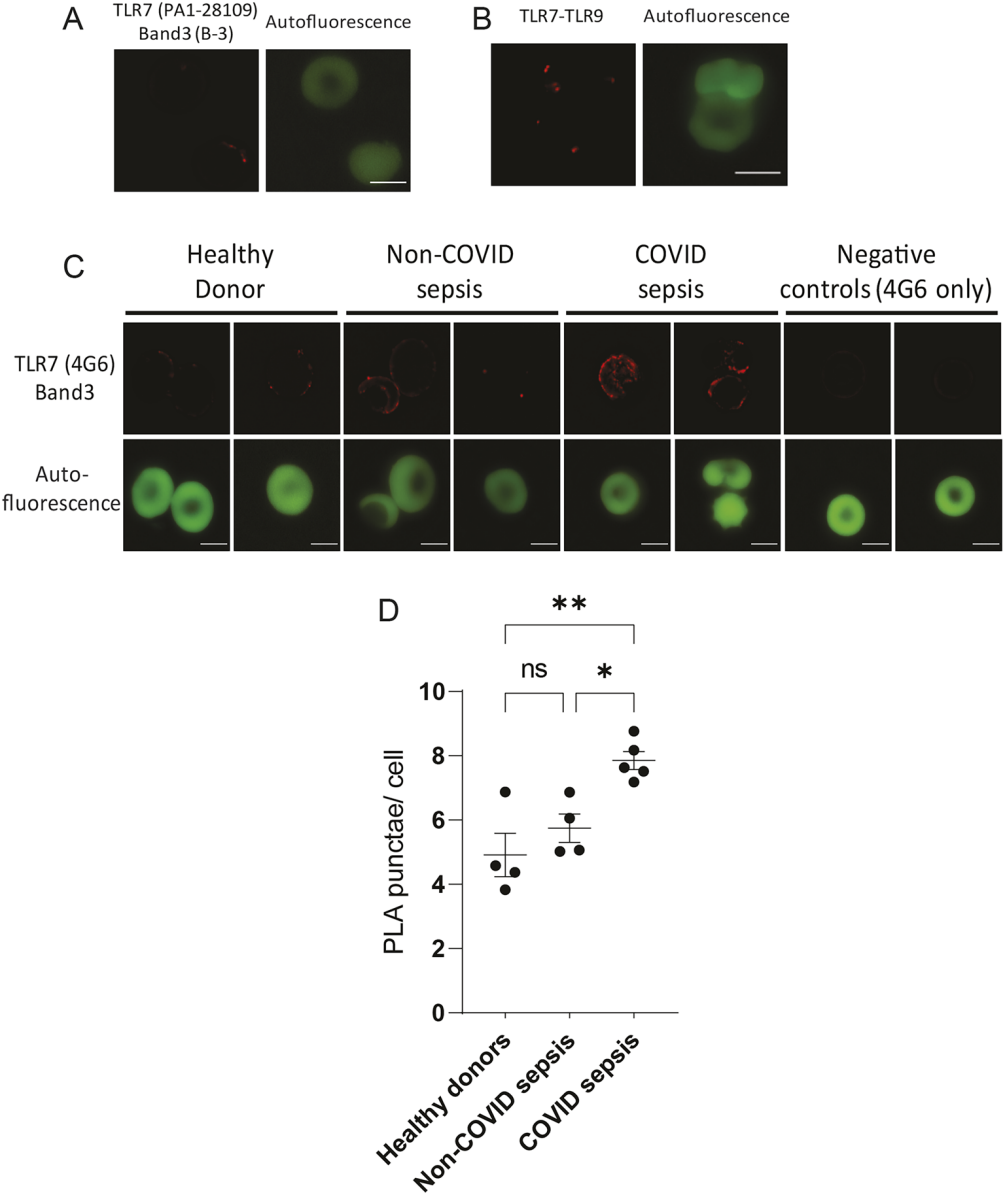
TLR7 is proximal to Band 3 on the RBC membrane (**A**) PLA for TLR7 (PA1-28,109) and Band 3 (A-6) and (**B**) PLA for TLR7 (4G6) and TLR9 (ab37154) on RBCs from a healthy donor. (**C**) PLA for TLR7 (4G6) and Band 3 (ab108414) on RBCs from healthy donors and sepsis patients, PLA signal (red) and autofluorescence of RBCs (green) are shown. Each pair of images represents a unique donor or patient, n = 13 donors, 8–9 independent experiments. (**D**) Quantification of PLA signals from (**C**). The difference between healthy donors and patients was evaluated by one-way ANOVA with Sidak’s multiple comparison, *P = 0.03, **P = 0.003. Scale bar represents 5 μm.

In order to determine the localization of TLR7 within the RBC, we next examined the interaction of TLR7 with the RBC membrane protein Band 3 via proximity ligation assay (Fig. 2A,B). Using RBCs purified from whole blood from healthy donors we found low levels of close interaction between TLR7 and Band 3 using two distinct antibody combinations (Fig. 2A,C). We also discovered TLR7 interacts with TLR9 (Fig. 2B), which interacts with Band 3 on the RBC membrane, although once again, very few PLA punctae were observed on RBCs from healthy donors^4,5^. Collectively, these data establish the presence of the RNA-sensing PRR TLR7 on human RBC membranes.

Band 3 alterations, including clustering and phosphorylation, are reported during inflammatory states, including sepsis and malaria^13–15^. Therefore, we asked if TLR7-Band 3 associations are increased in RBCs from patients with sepsis. While TLR7-Band 3 interactions in RBCs from healthy donors were detected by distinct PLA punctae, RBCs from patients with COVID-associated sepsis exhibited a robust PLA signal with aggregates of PLA punctae (Fig. 2C). This enhanced association was more prominent in viral sepsis due to SARS-CoV-2 infection when compared to non-COVID sepsis (Fig. 2C,D). Thus, TLR7 association with RBC membrane proteins is increased during SARS-CoV-2-induced sepsis.

### RBCs binds exogenous RNA

**Figure 3:**
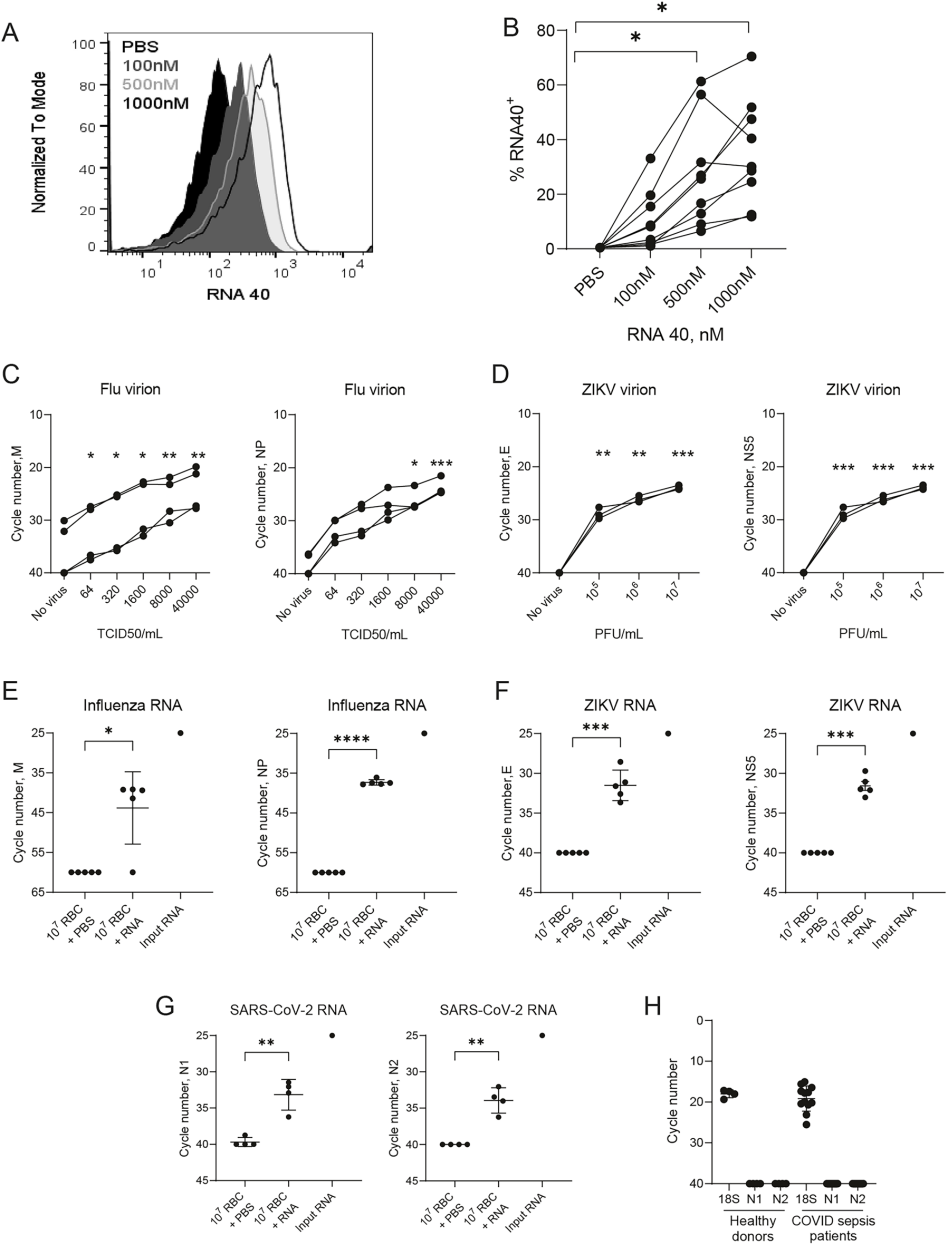
RBCs bind RNA (**A**,**B**) Binding of Cy5-RNA40 to RBCs from healthy donors. Representative histograms (**A**) and summarized data (**B**) are shown, n = 9, **P = 0.008 for 500 nM v PBS and **P = 0.002 for 1000 nM v PBS. (**C**,**D**) Binding of virus particles to RBCs. 10^7^ RBCs were incubated with indicated concentrations of influenza virus (**C**) or ZIKV (**D**) particles, and RBC-associated viral RNA was quantified with qRT-PCR. Amplicons for influenza matrix (M) and nucleoprotein (NP), and ZIKV envelope (E) and non-structural protein 5 (NS5) were used. (**E–G**) Viral RNA binding to RBCs. RBCs were incubated with 0.1 ng influenza virus RNA (**E**), 1 ng ZIKV RNA (**F**), or 1 ng SARS-CoV-2 RNA (**G**), and RBC-associated viral RNA was quantified with qRT-PCR. (**H**) RNA was extracted from RBCs from healthy donors and septic COVID patients and was quantified using qRT-PCR. N1 and N2 are different regions of the nucleocapsid gene of SARS-Cov-2, 18 s was used as an internal control. The limit of detection was set at 40 cycles (n = 12). One-way ANOVA with Dunnett’s post-hoc test was used in (**B**–**D**), and t-test was used in (**E**–**G**) *P < 0.05; **P < 0.01; ***P < 0.005.

We next asked whether RBCs bind exogenous RNA. We found dose-dependent binding of a Cy5-labeled ssRNA oligoribonucleotide (RNA40) to healthy donor RBCs (Fig. 3A,B)^16^. Because the acquisition of DNA by RBCs led to masking of the anti-phagocytic epitope of the marker of self, CD47, we asked whether RNA binding would elicit the same response in RBCs^5^. RNA treatment of RBCs did not mask the CD47 anti-phagocytic epitope, suggesting that RNA and DNA have distinct effects on RBCs (Supplemental Fig. 2A,B). We also asked whether RNA binding by RBCs would increase exposure of the senescent protein phosphotidylserine (PS), on the RBCs. RNA-40 acquisition did not increase PS exposure on RBCS (Supplemental Fig. 2C). Collectively, these data demonstrate that ssRNA acquisition by RBCs does not accelerate RBC senescence.

We next asked whether RBCs acquire pathogen-derived RNA. We examined RNA-acquisition by RBCs following incubation of RBCs with RNA viruses known to bind RBCs. Because influenza virus binds directly to RBCs through hemagglutinin-sialic acid interaction^17,18^, we first asked whether viral RNA would be acquired by RBCs following incubation with influenza virus (A/Puerto Rico/8/1934 H1N1). We performed qPCR for influenza virus RNA following incubation of RBCs with increasing doses of influenza virions. Influenza virus RNA is detectable in RBCs following incubation of influenza virions with naive RBCs (Fig. 3C), indicating that RBCs acquire viral RNA following binding to virions. Zika Virus (ZIKV) is a mosquito-borne flavivirus that may persist in the circulation for weeks after infection^19^. It has recently been reported that ZIKV is detected in the RBC fraction of transfusates, yet it is unknown if ZIKV directly binds to RBCs or infects erythroid precursors^20^. We found that naive, mature RBCs bind ZIKV (Fig. 3D). We next asked whether RBCs acquire viral RNA by directly binding to RNA; we incubated RBCs with RNA extracted from influenza A virions, Zika virions, or SARS-CoV-2 RNA (Fig. 3E–G). Although we observed heterogeneity in the ability of human RBCs to bind viral RNA, viral RNA adhered to RBCs, suggesting that RBCs are capable of directly binding RNA from viruses.

Given recent reports of direct infection of erythroid progenitors with SARS-CoV-2 and our own findings of RBC-bound SARS-CoV-2 spike protein^21,22^, we evaluated RBCs from patients hospitalized with COVID-19 (Fig. 3H). We did not detect SARS-CoV-2 on RBCs from patients with COVID-19.

### RNA binding is attenuated with TLR7 inhibtors

**Figure 4:**
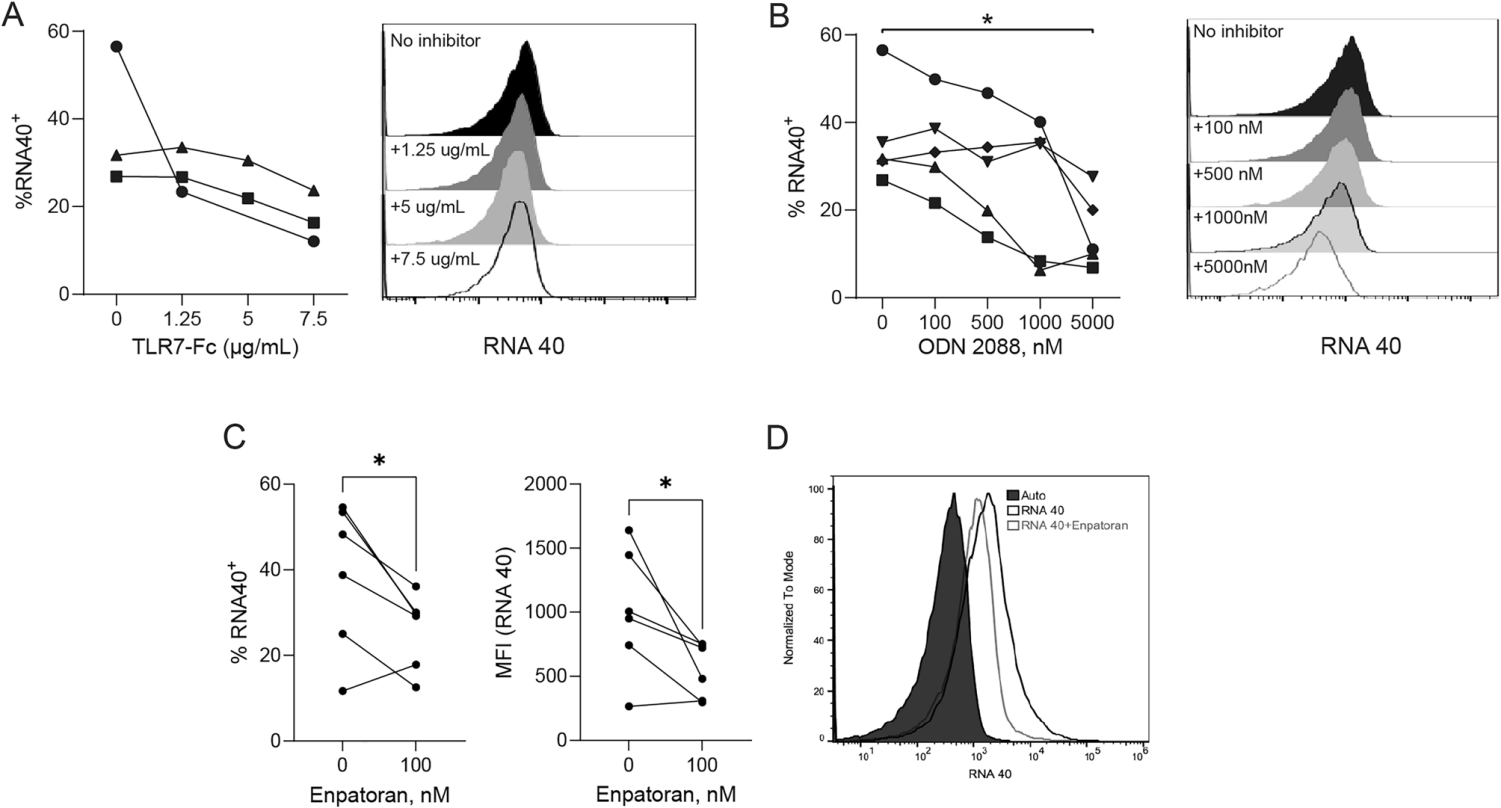
ODN 2088 and Enpatoran attenuate RBC-RNA acquisition (**A**) Binding of RNA40 to RBCs in the presence of TLR7-Fc. Percent RNA40^+^ cells and a representative histogram are shown. (**B**) RNA40 binding to RBCs in the presence of ODN2088. Percent RNA^+^ cells and a representative histogram are shown. *P = 0.018, *P* = 0.032 (for 0 nM v 5000 nM), one-way ANOVA with Sidak’s multiple comparison. (**C**) RNA40 binding to RBCs in the presence of Enpatoran (100 nM), percent RNA^+^ cells and MFI are shown, *P = 0.039 for RNA40 + cells and *P = 0.046 for MFI. (**D**) Representative histogram, for **C** and **D**, n = 6 healthy donors, each line dot pair represents an individual donor.

We next asked if blocking ssRNA binding with TLR7-Fc, inhibitory ODNs, or Enpatoran (a selective TLR7/8 inhibitor) would attenuate ssRNA acquisition by RBCs. RNA40 binding to RBCs was examined in the presence of increasing doses of soluble recombinant human TLR7-Fc. We observed inhibition of RNA-40 binding to RBCs in only one of three donors tested (Fig. 4A). We then asked if inhibitory oligonucleotides could prevent RNA binding by RBCs. ODN 2088 and ODN 20959 are CpG-containing oligonucleotides that inhibit TLR7 and TLR9 responses in human and murine myeloid cells, whereas ODN 105870 is derived from ODN 20959 but contains a modified guanine that renders it inhibitory to TLR7, not TLR9^23,24^. We observed a dose-dependent inhibition of RNA40 binding by RBCs in the presence of ODN 2088 (Fig. 4B), although we observed substantial heterogeneity in the inhibition of RNA binding by ODN 2088 amongst donors. However attenuation of RBC-RNA binding by ODN20959 and ODN105870 was observed in only one donor (Supplemental Fig. 3A,B), and we did not observe statistically significant inhibition of RNA-40 binding to RBCs with these ODNs. We next tested the ability of ODN to inhibit viral RNA acquisition by RBCs and found that ODN 2088 attenuated pathogen-derived viral RNA acquisition (Supplemental Fig. 3C,D). Because ODN 2088 is an inhibitor of TLR7 and TLR9 and is not specific for TLR7 we next asked whether RNA40 acquisition by RBCs would be attenuated in the presence of Enpatoran, a small molecule TLR7/8 inhibitor. The addition of Enpatoran attenuated the uptake of RNA40 by RBCs (Fig. 4C,D). Collectively, these data suggest that RBCs bind synthetic ssRNA which can be attenuated in the presence of the TLR7 inhibitor Enpatoran.

## Discussion

We demonstrate that TLR7 is expressed on RBCs and observe increased membrane association of TLR7 during infection. Moreover, we show that RBCs can acquire RNA from pathogenic viruses and that RNA acquisition is attenuated by the TLR7/8 inhbitor Enpatoran. To our knowledge, these findings represent the first report of an RNA sensor on RBCs and suggests additional immune functions of the RBC that have yet to be elucidated.

We observed TLR7- Band 3 proximity that was enhanced in patients with COVID-associated sepsis when compared with non-COVID septic patients and healthy control subjects. We also observed TLR7-TLR9 proximity in the RBC. The association of TLR7, TLR9, and Band 3 on the RBC surface during viral infection suggests a potential immune receptor complex on the RBC. Band 3 is an anion exchanger expressed on the RBC surface that forms various multi-protein macro complexes to maintain optimal RBC structure and function^25,26^. Clustering proteins of similar function in close proximity is reminiscent of cellular membrane lipid rafts where molecules of related functions are concentrated to facilitate efficient signaling and regulation. Overlapping and interacting TLR signaling cascades have previously been demonstrated in pure immune cell populations, where TLR clustering during infection may serve to amplify downstream inflammatory responses. However, we have not observed cell-intrinsic signaling in RBCs following DNA-binding and RNA binding did not increase RBC senescence as measured by loss of CD47 or PS externalization^5^. Therefore, we do not believe that the TLRs signal within the RBC but act as scavenger receptors to bind nucleotides.

We demonstrate that RBCs are able to bind synthetically created RNA sequences, but also able to retain RNA from known RBC-binding viruses including influenza, ZIKA, and SARS-CoV2^27–29^. Dating back to the HIV pandemic, transfusion-associated viral infection has long been a concern in blood banking. While the advent of molecular screening and nucleic acid amplification techniques has dramatically reduced the risk of viral infection following blood transfusion, one lingering question has been the degree of persistence of the virus in transfusates^30^. RBC-associated West Nile virus (WNV) virions are reported in transfusates^31^, and persistence of RBC-associated ZIKV RNA has been reported in asymptomatic patients^27^, prompting the question of whether RBCs can sequester RNA from tissues and the circulation, possibly after the live virus has been cleared, serving to prevent host immune responses to viral RNA. Supporting this hypothesis are reports, demonstrating that nucleic acids trapped in RBCs are supposedly nuclease resistant^32^. In light of this evidence, it is plausible that RBC-associated RNAs are protected from degradation and hence detected in banked blood, even if the donor no longer harbors the infectious virus.

By employing the inhibitory ODN 2088, we observed a reduction of RNA acquisition by RBCs. This inhibition of RNA binding by RBCs was further validated using the selective TLR7/8 inhibitor, Enpatoran. However, additional investigations are warranted to establish the direct binding of single-stranded RNA (ssRNA) to TLR7 on RBCs. Nevertheless, our study confirms the ability of RBCs to acquire exogenous TLR7 ligands, suggesting an additional role in sequestering ssRNA. However, the impact of RBC-RNA binding on either dampening or enhancing the host inflammatory response to TLR7 ligands derived from pathogens or the host itself has yet to be fully elucidated.

In our experiments, fixation and detergent treatment were required for epitope accessibility of both TLR7 antibodies (4G6 and PA1-10826) raised against the TLR7 ectodomain; however, the proximal interactions of TLR7 and Band 3 and susceptibility of RNA binding to TLR7 inhibitory oligonucleotides suggest TLR7 is localized on the RBC plasma membrane. Indeed we were able to detect low levels of TLR7 on the RBC surface using an antibody previously shown to detect TLR7 on the surface of murine cells^3^. However, these findings will need be validated in larger cohorts. Using the TLR7 antibody (A94B10) for RBC surface staining, we were able to detect a low level of surface TLR7 expression that appears to be heterogeneous across healthy donors. Because the structure of Band 3 changes as RBCs age and during inflammatory conditions, it will also be important to determine if TLR7 adopts a non-canonical topology on stressed RBC membranes or if our findings of increased Band 3-TLR7 proximity in critically ill patients with COVID-associated sepsis is due to enhanced epitope accessibility. Indeed, it has previously been demonstrated that the RBC membrane is altered during sepsis and several studies have demonstrated altered RBC rheology during COVID-19 infection^33–35^.

In conclusion, our collective data affirm the expression of the immune receptor TLR7 on RBCs and their capability to bind RNA. The ability of RBCs to capture exogenous RNA suggests that mature, enucleated RBCs can function as an RNA scavenger or reservoir within the circulation. Future work examining the characteristics of RBC-associated RNA during inflammation and the role of immune receptor expression on RBCs will be needed in order to address our knowledge gap in the long-ignored non-gas exchanging immune function of RBCs.

## Methods

### Study approval for healthy adults

Studies involving human subjects were approved by the University of Pennsylvania Institutional Review Board. Healthy volunteers between 18 and 65 years gave written informed consent before inclusion.

### Sepsis cohort

RBCs were obtained from patients enrolled in the Molecular Epidemiology of Severe Sepsis in the ICU (MESSI) cohort or inpatient subjects positive for SARS-CoV-2 enrolled in the MESSI-COVID study at the University of Pennsylvania; inclusion and exclusion criteria for the MESSI and MESSI-COVID cohorts were previously reported^36,37^. Human subjects or their proxies provided informed consent. All human subjects studies were performed in agreement with the Declaration of Helsinki.

### Antibodies

TLR7 was detected with 4G6 (Novus Biological, FITC-conjugated at 5 μg/assay or unconjugated at 2.5 μg/mL), PA1-28,109 (Invitrogen, 5 μg/mL), or A94B10 (Biolegend, 5ug/mL). TLR9 was detected with ab37154 (Abcam, 5 μg/mL). Band 3 was detected with ab108414 (Abcam, 1.4 μg/mL) or A-6/sc 133,190 (Santa Cruz, 1 μg/mL). Antibodies against CD41 (HIP8, APC-conjugated, 7.5 μg/mL), GPA (HI264, PE-conjugated, 0.5 μg/mL), and CD47 (CC2C6, APC-conjugated, 5 μL/assay) were purchased from Biolegend.

### Oligonucleotides

Oligonucleotides were purchased from IDT. The sequences are:

RNA40-Cy5 or FAM:

rG*rC*rC*rC*rG*rU*rC*rU*rG*rU*rU*rG*rU*rG*rU*rG*rA*rC*rU*rC-Cy5 or 6-FAM;

ODN2088: T*C*C*T*G*G*C*G*G*G*G*A*A*G*T;

ODN20959: T*A*A*T*G*G*C*G*G*G*G*A*A*G*T;

ODN105870: T*A*A*T*G*G*C*E*G*G*G*A*A*G*T, where “r” denotes ribonucleotide, “*” denotes phosphorothioate bond, and “E” denotes 7-deaza-2′-deoxyguanosine.

### RBC isolation

Whole blood was centrifuged at 3000 g for 10 min. The plasma and buffy coat were removed and frozen. RBCs were purified from the remaining packed red cell fraction using MACS or leukoreduction filters as previously described^4,5^. MACS-isolated RBCs were fixed for staining (see below) or frozen at − 80 °C for qPCR. To detect SARS-CoV-2 RNA, 5 μL of packed RBCs from healthy donors or patients were frozen after centrifugation.

### Flow cytometry

For surface staining, 250,000 cells were washed with and blocked in anti-human Fc block, followed by staining with CD45, CD41, and GPA Abs diluted in FACS buffer (PBS + 2% FBS) for 30 min on ice. For TLR7 staining, cells were washed three times in PBS, fixed with 0.05% glutaraldehyde in PBS for 10 min at room temperature, washed in FACS buffer, and permeabilized in 0.1% Triton X-100 diluted in FACS buffer for 15 min. After three washes, cells were stained with FITC-conjugated anti-TLR7 Ab, 4G6, or isotype for 1 h. Cells were washed twice before analysis (BD Fortessa and FlowJo). For surface TLR7 staining, cells were washed with PBS and stained with PE-conjugated anti TLR7 Ab, clone A94B10, for one hour at room temperature. Cells were washed twice then analyzed (Cytoflex and FlowJo).

### Immunofluorescence and microscopy

RBCs were fixed and permeabilized as above. RBCs were blocked in PBST (PBS + 0.05% tween20) supplemented with 1% BSA and 5% goat serum for 1 h at room temperature. 10^6^ fixed, permeabilized, and blocked RBCs were stained with primary Abs mentioned above diluted in PBST with 1% BSA overnight at 4 °C. Cells were washed in PBST and stained in secondary Abs raised in goat (Jackson ImmunoResearch) for 1 h at room temperature. After washing, RBCs were resuspended in PBS and mounted with Fluoromount G. Confocal micrographs were acquired with a VT-iSIM (Visitech).

### Proximity ligation assay

(PLA, DuoLink, Sigma) was carried out according to the manufacturer’s protocol. Stained cells were resuspended in 10 μL PBS and mounted on Fluoromount G. Images were acquired with a Nikon 2A microscope. At least five fields were imaged for each sample, and the number of PLA punctae per cell were counted by two blinded personnel.

### RNA extraction and reverse transcription

RNA was extracted from frozen RBCs using the RNeasy Plus Kit (Qiagen). At the final step, RNA was eluted in 30 μL RNase-free water, and 8 μL of freshly isolated RNA was reverse transcribed to cDNA using the Superscript First Strand Synthesis System (ThermoFisher).

### qRT-PCR

The QuantStudio7 Flex system (Applied Biosystems) was used to perform qRT-PCR. TaqMan Fast Universal master mix was used to detect CD41 (TaqMan assay Hs01116228_m1) and SARS-CoV-2 RNA (IDT, 2019-nCoV RUO Kit). Zika Virus (ZIKV) and Influenza RNA were detected using the PowerUp SYBR Green master mix and primers listed below.

ZIKV-E: 5′-TTGGTCATGATACTGCTGATTGC-3′ and 5′-CCTTCCACAAAGTCCCTATTG C-3′. ZIKV-NS5: 5′-GGCCACGAGTCTGTACCAAA-3′ and 5′-AGCTTCACTGCAGTCTTC C-3′. Influenza-M: 5′-GGACTGCAGCGTAGACGCTT-3′ and 5′-CATCCTGTTGTATATGAGGCC CAT-3′. Influenza-NP: 5′-GACGATGCAACGGCTGGTCTG-3′ and 5′-ACCATTGTTCCAACT CCTTT-3′.

### Oligoribonucleotide (ORN) binding and inhibition

250,000 RBCs resuspended in 100 μL sterile PBS were mixed with Cy5-labeled ORN (RNA40) in polypropylene tubes. In assays where inhibitors were tested, 500 nM RNA40 was incubated with RBCs in the presence of the specified concentrations of inhibitors. Recombinant human TLR7-Fc was obtained from R&D Systems and Enpatoran from Invivogen. The tubes were then sealed in Parafilm and incubated at 37 °C for 2 h on a nutator in the dark. Following inhibition with Enpatoran, cells were washed, resuspended in PBS, and incubated with live/dead aqua stain (Invitrogen) at room temperature for 30 min on a nutator protected from light. Cells were washed with PBS and analyzed by flow cytometry.

### CD47 and Annexin V staining

RBCs incubated with RNA40 for 2 h were washed with FACS buffer twice and probed with 5 μg anti-CD47 Ab (CC2C6, Biolegend) for 1 h. Cells were then washed with FACS buffer three times and analyzed by flow cytometry. For assessment of Phosphotidylserine externalization, RBCs were stained for Annexin V (Life Technologies) as we previously described^5^.

### Virion and viral RNA binding

Virus stocks and RNA were provided by Dr. Andrew Vaughn (Influenza virus) and Dr. Kellie Jurado (Zika virus and SARS-CoV-2). All viral particle and RNA binding assays were performed in a 100 μL reaction volume in DNA or RNA lo-bind tubes (Eppendorf, 86-924). All viruses and corresponding RNAs were diluted in sterile PBS. 10^7^ RBCs were used in viral particle binding assays. For RNA binding inhibition, 1 ng viral RNA and 10^7^ RBCs were used. RBCs were incubated with virus particles or RNA for 2 h at 37 °C on a nutator. The tubes were rotated at 1 h to ensure suspension of cells. For virus particle binding, RBCs were washed with PBS three times and frozen until RNA extraction. For viral RNA binding assays, the RNA-RBCs mixture was overlaid on 500 μL 30% sucrose in PBS at 4 °C. Cells were pelleted by centrifugation at 13,000 rpm for 3 min, followed by two additional washes in PBS, and frozen until RNA extraction.

### Supplementary Information

Supplementary Figures.

## Data Availability

All data used in the current study will be made available from the corresponding author upon request.
