# Supplementary material for: Human red blood cells express the RNA sensor TLR7
Source: Sci Rep. 2024 Jul 9;14:15789. doi: 10.1038/s41598-024-66410-5 (PMC11233670; doi:10.1038/s41598-024-66410-5)
Supplement: Supplementary file 1 — Supplementary Figures. [file 41598_2024_66410_MOESM1_ESM.pdf]

Human red blood cells express the RNA sensor TLR7

LK Metthew Lam[1], Emily Oatman[2],Kaitlyn A. Eckart[1], Nathan J. Klingensmith[2],Emily Flowers[5] Layal Sayegh[1], Julia Yuen[1], Rebecca L. Clements[3], Nuala J. Meyer[1], Kellie A. Jurado[3], Andrew E. Vaughan[4], Stephanie C. Eisenbarth[5], Nilam S. Mangalmurti[1]

[1]Division of Pulmonary, Allergy, and Critical Care, Perelman School of Medicine at the University of Pennsylvania, Philadelphia, PA 19104, USA

[2] Division of Traumatology, Surgical Critical Care, and Emergency Surgical Services, Department of Surgery, Perelman School of Medicine at the University of Pennsylvania, Philadelphia, PA 19104, USA

[3] Department of Microbiology, University of Pennsylvania, Philadelphia, PA, 19104, USA

[4] Department of Biomedical Sciences, University of Pennsylvania School of Veterinary Medicine, Philadelphia, PA 19104, USA

[5]Department Medicine, Division of Allergy and Immunology, Northwestern University Feinberg School of Medicine, Chicago, IL 60611, USA

Corresponding author:

Nilam S. Mangalmurti

215-746-0376 (fax)

215-573-4590 (office)

nspatel@pennmedicine.upenn.edu

Running title: Human red blood cells express TLR7

Keywords: Red blood cell, toll-like receptor, TLR7, RNA

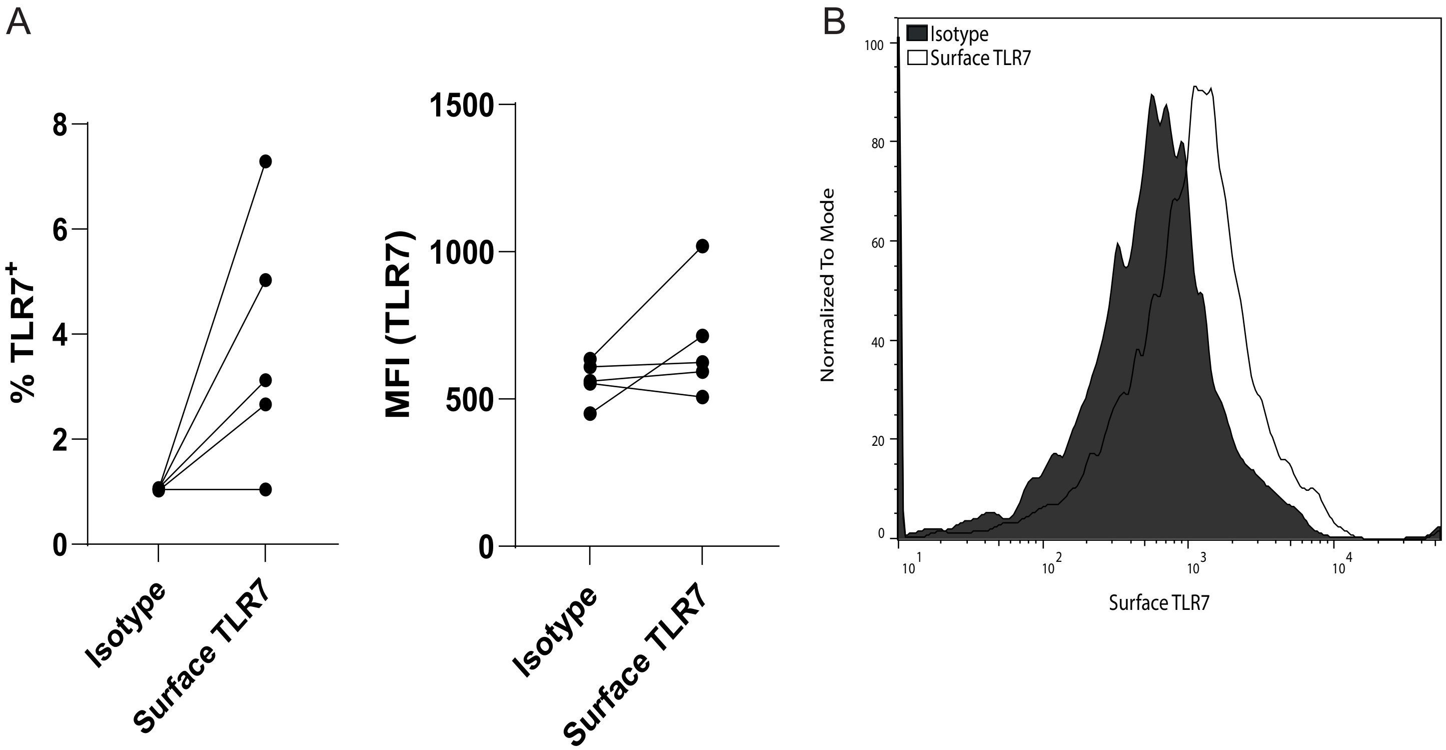

Supplemental figure 1. RBC surface TLR7 is detectable in low amounts. Surface TLR7 detected by flow cytometry on RBCs from healthy donors (n=5). (**A**) Percentage of surface TLR7 detected on RBCs compared to isotype-stained controls, P=0.06, paired t test. MFI of surface RBC TLR7 or isotype-stained controls, P=0.19, paired t test. (**B**) Representative histogram from a single healthy donor.

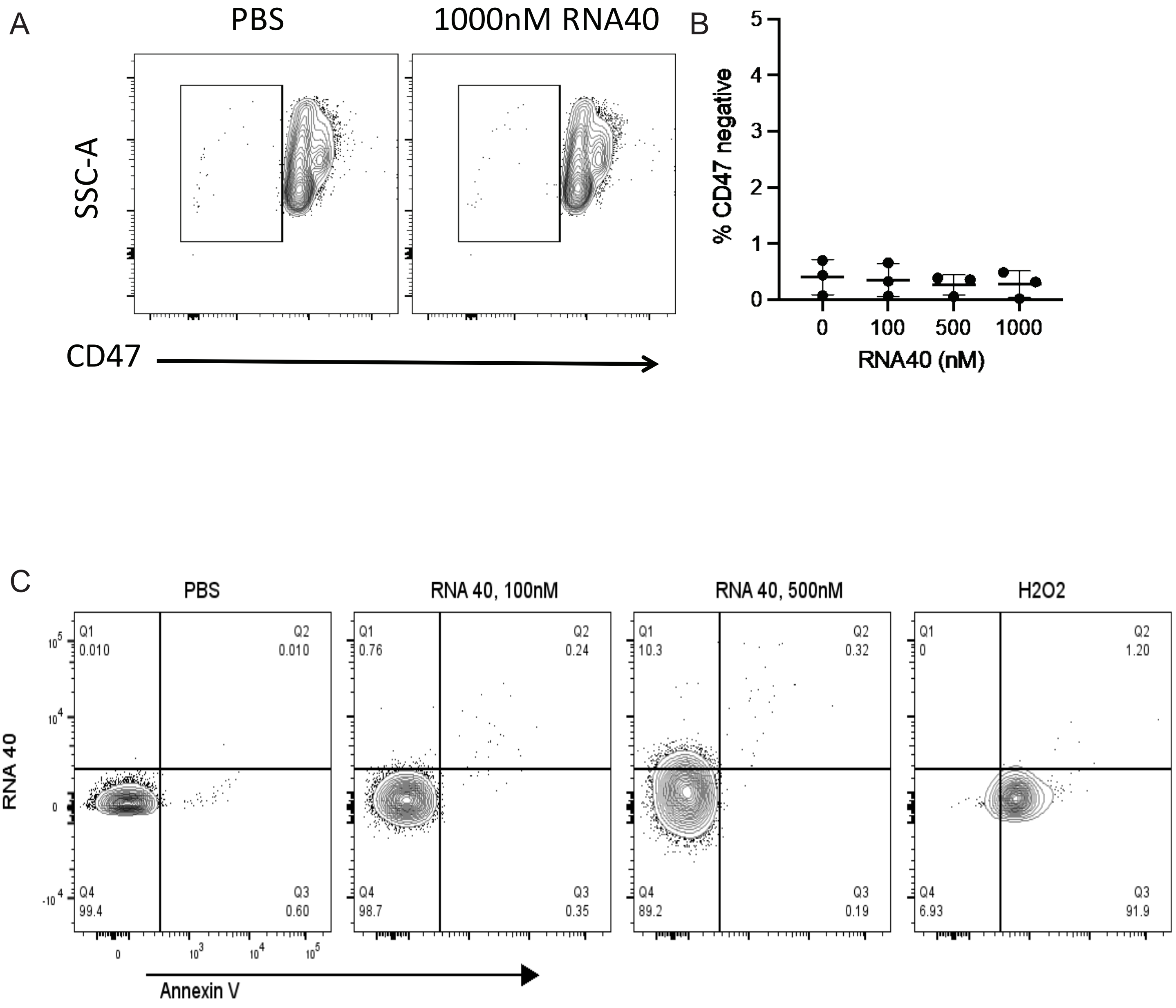

Supplemental figure 2 . RNA40 acquisition does not result in loss of CD47 or PS externalization. CD47 expression on RNA40 treated RBCs from healthy donors. **(A)** A representative contour plot and **(B)** summary of the data for individual donors is shown, n=3. **(C)** Annexin V staining on RNA40-treated RBCs. H2O2-treated RBCs are shown as a positive control.

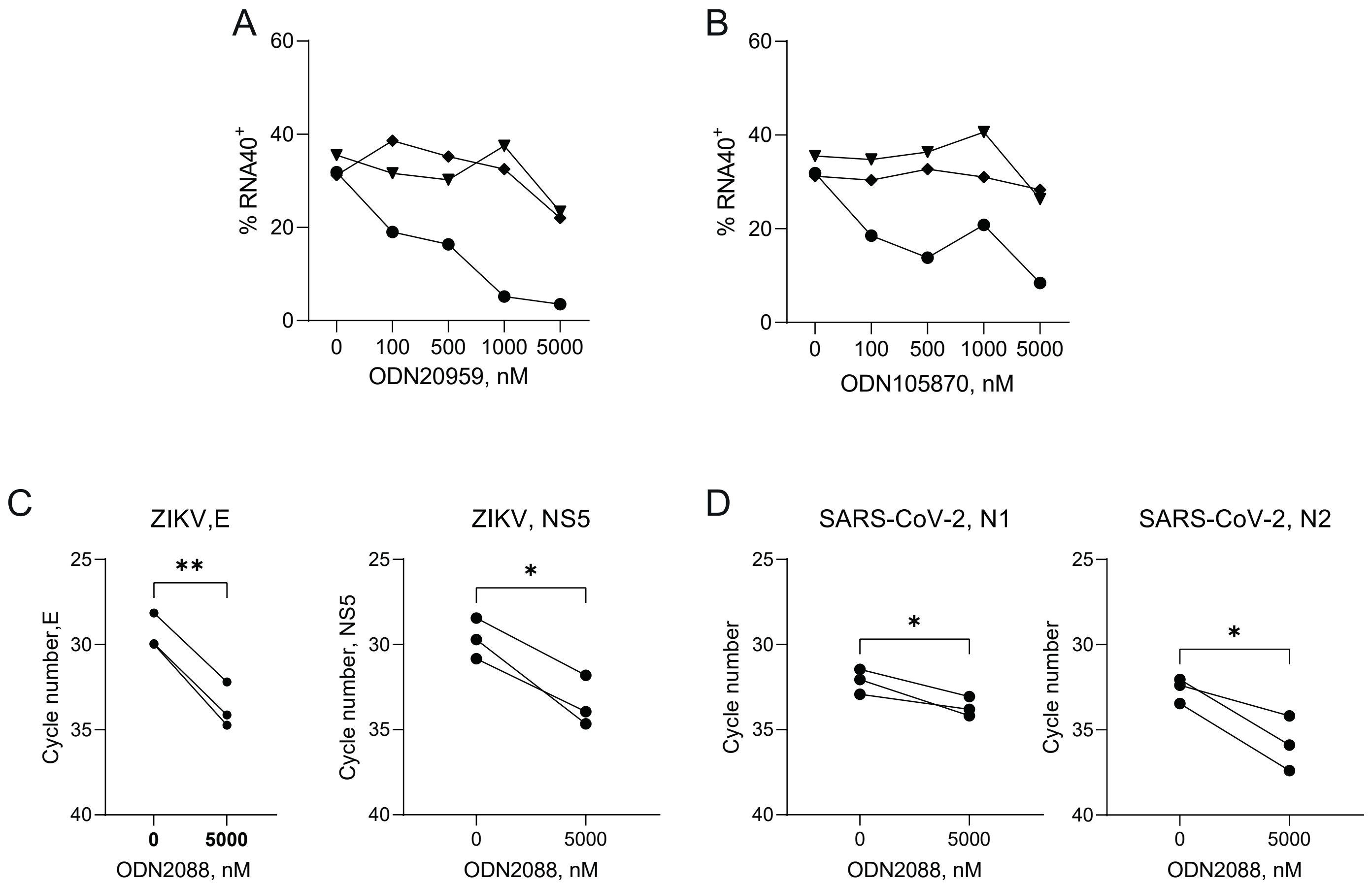

Supplemental figure 3. Attenuation of RNA acquisition in the presence of inhibitory ODNs. (A) Binding of RNA 40 to RBCs in the presence of ODNs 20959 and **(B)**105870. Quantification of RBC-associated viral RNA upon incubation of ZIKV **(C)** or SARS-CoV-2 **(D)** viral RNA with RBCs in the presence of 5000nM ODN2088, * $P<0.05$; ** $P<0.01$, paired t-test. For A and B, each symbol line pair represents an individual donor, n= 3 healthy donors. For C and D, each circle-line pair represents an individual donor, n=3 donors.
